# Editorial: Simultaneous EEG-fMRI applications in cognitive neuroscience

**DOI:** 10.3389/fnhum.2023.1350468

**Published:** 2024-01-04

**Authors:** Elias Ebrahimzadeh, Hamid Soltanian-Zadeh

**Affiliations:** ^1^Control and Intelligent Processing Center of Excellence (CIPCE), School of Electrical and Computer Engineering, College of Engineering, University of Tehran, Tehran, Iran; ^2^School of Cognitive Sciences, Institute for Research in Fundamental Sciences (IPM), Tehran, Iran

**Keywords:** simultaneous EEG-MRI, cognitive neuroscience, functional magnetic resonance imaging (fMRI), electroencephalography (EEG), decision-making

## Introduction

Simultaneous electroencephalography (EEG) and functional magnetic resonance imaging (fMRI) is a complementary technique that was idealized as a tool to precisely localize regions in the brain associated with scalp EEG findings. The classical method evaluates blood oxygenation changes in fMRI temporally correlated with an electrocortical event, taking advantages from high spatial resolution of fMRI and high temporal resolution of EEG.

Applications of EEG-fMRI include research in basic cognitive function and its dynamics, decision-making, sleep, resting-state networks, neurofeedback, emotion foci localization, and especially epilepsy. Recent studies have illustrated the value of EEG-fMRI for localization of neural networks, and the application of the technique as a complementary tool for identification of brain regions involved in different tasks and conditions is increasing. For example, since mono-module techniques do not show completely consistent brain activation of regions related to feedback stimuli, recent studies have adopted EEG-fMRI fusion technology to reveal brain neural activity in this condition.

The goal of this Research Topic is to expand applications of EEG-fMRI and advance the methods from preprocessing to event detection and analysis. We aim to improve the understanding of the relation between cognitive processes and resting state networks, dynamics of cognitive processes, and applications of machine learning methodologies on the EEG or fMRI data and the relationship between findings. Methods and applications in cognitive neuroscience using EEG-fMRI aims to highlight the latest experimental techniques and methods used to investigate fundamental questions about the mental processes involved in cognition.

## Research Topic coverage

This Research Topic includes two reviews, five research articles, and a hypothesis and theory article. The accepted articles cover simultaneous EEG-fMRI processing for specific applications, a novel model for EEG and fMRI performance in cognitive neuroscience, or the identification of areas involved in cognitive tasks using simultaneous EEG-fMRI acquisition.

Neurofeedback emotion regulation by functional connectivity analysis is among the topics covered in this Research Topic. The article entitled “*Dynamic functional connectivity estimation for neurofeedback emotion regulation paradigm with simultaneous EEG-fMRI analysis*” by Mosayebi et al. investigates simultaneous EEG-fMRI data on a self-regulation neurofeedback paradigm. They have employed the recently introduced Correlated Coupled Tensor Matrix Factorization (CCMTF) method for analysis of the emotion regulation paradigm based on the EEG frontal asymmetry neurofeedback in the alpha frequency band with simultaneous fMRI. They found the CCMTF method a capable approach for extracting the information shared between the EEG and the fMRI data, revealing new information about the brain functions and their connectivity without solving the EEG inverse problem or analyzing different frequency bands.

Nili et al. have conducted a systematic review covering the current state-of-the-art in the variation of functional connectivity and activity before and after thalamotomy surgery. In this review, they have summarized the use of fMRI and EEG in thalamotomy surgeries. Their analysis shows that thalamotomy surgery can result in changes in functional connectivity in motor-related, visuomotor, and default-mode networks, as detected by fMRI. The EEG data also indicate a reduction in the over-activities observed preoperatively.

Li et al. have proposed a source localization method for the multimodal integration of the EEG and fMRI data and tested it in the source-level functional network analysis of emotion cognitive reappraisal. The proposed multimodal integration method for source localization has identified the key cortices involved in emotion regulation, and the network analysis has demonstrated the important brain regions involved in the cognitive control of reappraisal. It shows promise in the utility in the clinical setting for affective disorders.

Jacob et al. have suggested that by combining EEG with fMRI, new experimental work can reconcile emerging controversies in neurovascular coupling and the significance of ongoing, background activity during resting-state paradigms. In this article, a new conceptual framework for neuroimaging paradigms is developed to investigate how ongoing neural activity is “entangled” with metabolism. This framework demonstrates how multimodal neuroimaging is necessary to probe the neurometabolic foundations of cognition, with implications for the study of neuropsychiatric disorders.

Dehghani et al. have presented a novel approach that deals with EEG neurofeedback (NF) with simultaneous fMRI using a modified happiness-inducing task through autobiographical memories to upregulate positive emotion. The results reveal several new connections among the brain regions as a result of EEG neurofeedback, which can be justified according to emotion regulation models and the role of those regions in emotion regulation and recalling positive autobiographical memories.

Ciccarelli et al. have conducted a systematic review of EEG-fMRI-NF studies (*N* = 17) to identify the potential and effectiveness of this non-invasive treatment for neurological conditions. The systematic review has revealed a lack of homogeneity among the studies, including sample sizes, acquisition methods in terms of simultaneity of the two procedures (unimodal EEG-NF and fMRI-NF), therapeutic target fields, and the number of sessions.

Yousefi et al. have proposed a novel deep-learning approach for emotion recognition that encompasses multiple stages, including feature extraction and feature selection, as well as clustering and classification using Dual-LSTM (long short-term memory). This method can be useful in various applications, such as developing more effective therapies for individuals with mood disorders or improving human-computer interaction by allowing machines to respond more intelligently to users' emotional states.

Epilepsy is a neural disorder that, if diagnosed early, can prevent injuries to the patient. The article entitled “*Detection of epileptic seizures through EEG signals using entropy features and ensemble learning*” by Dastgoshadeh and Rabiei has presented a new method for automatic detection of epileptic seizures using entropy-based features and ensemble learning. The results have shown that the proposed method is highly accurate in detecting epileptic seizures; it offers improvement over most similar methods and can be used as an effective tool in diagnosing this complication.

All of the articles have presented important ideas for the identification of the brain regions and the neural networks involved in different tasks or conditions through the acquisition of the simultaneous EEG and fMRI data and multi-modality processing of the acquired data.

The editors are pleased to present this Research Topic of articles to the field of EEG-fMRI integration in cognitive neuroscience and the related scientific communities. They hope that their Research Topic is found interesting to the readers of the articles and the researchers working in the fields of medicine, biomedical engineering, and neuroscience.

## Author contributions

EE: Conceptualization, Writing—original draft. HS-Z: Supervision, Writing—review & editing.

